# Impact of Sprint Performance Characteristics Across Acceleration–Initial Speed Profiles in LALIGA

**DOI:** 10.3390/sports14070273

**Published:** 2026-07-01

**Authors:** José Luis Quintero-Illera, Fabio Nevado Garrosa, Raúl Zarzuela-Martin, Roberto López-Del Campo, Víctor Cuadrado-Peñafiel

**Affiliations:** 1 Department of Physical Education, Sport and Human Motricity, Autonomous University of Madrid, 28049 Madrid, Spain; victor.cuadrado@uam.es; 2 Football Intelligence and Performance, LALIGA, 28043 Madrid, Spain; 3 Department of Health Sciences, Miguel de Cervantes European University, 47012 Valladolid, Spain

**Keywords:** acceleration, initial velocity, physical performance, playing position, soccer

## Abstract

The aims of the present study were to (a) analyze the relationship between acceleration capacity at low and high initial running speeds and the player’s ability to cover high-intensity running distances and perform high-intensity accelerations during competition and (b) examine whether these differences also occur across playing positions in professional soccer players. A total of 222 professional male soccer players from LALIGA participated in the study during the 2021–2022 and 2022–2023 seasons. Players were classified in six positions: central defender (CD), full back (FB), central midfielder (CM), offensive midfielder (OM), winger (W) and forward (F). K-means clustering was applied to classify players based on the maximal theoretical acceleration (A_0Int_) capacity and the maximal theoretical initial running speed (S_0Int_). Mixed models were used to examine the accelerations performed above 75% of the acceleration–speed profile (Mean_75_AS_0_) according to IntS; distance covered at high speeds (21–24 km·h^−1^, >24 km·h^−1^, >28 km·h^−1^); and maximum speed across playing positions, cluster groups and contextual variables. C1 (high A_0Int_ and S_0Int_) showed a significantly greater distance covered during high-intensity running and accelerations than C2 (low A_0Int_ and S_0Int_) across all IntS thresholds. C3 (high A_0Int_ and low S_0Int_) achieved higher accelerations at a low IntS. In conclusion, offensive positions displayed greater distance covered during high-intensity running and acceleration across all IntS categories, while CD produced lower values. In conclusion, this study highlights how the accelerations quantified in relation to the IntS vary according to the relationship between A_0Int_ and S_0Int_ during competition and the positional role of the player.

## 1. Introduction

Soccer is an intermittent sport characterized by a prevalence of low-intensity activity and long rest periods, interspersed with brief but demanding bouts of high-intensity actions (HIAs) [1], such as sprints, accelerations, decelerations and multidirectional efforts, which are key physical parameters for optimal performance. These HIAs are typically associated with determinant offensive and defensive actions, such as goal scoring, creating goal-scoring opportunities, producing shots on target, making physical efforts to prevent counterattacks, and defending the goal [2,3].

Over recent years, global positioning systems and optical video tracking systems have been increasingly adopted in elite soccer competitions. Their implementation has enabled the systematic monitoring of large cohorts of professional soccer players, proving valuable insights into physical performance [4,5,6], positional-specific demands [7] and the evolving nature of competitive requirements [8]. Consequently, researchers have focused on developing novel methodologies to evaluate and enhance sprint performance during match play. These approaches aim to characterize HIAs through acceleration profiles, which have a significant impact on mechanical load and impose a high metabolic cost on players (e.g., accelerations, decelerations and direction changes), as well as through speed-based profiles associated with high-speed running (e.g., sprint actions above 24 km·h^−1^ and total distance covered above 24 km·h^−1^) [9,10,11]. However, HIAs typically occur over distances shorter than 20 m [12], highlighting the importance for elite players to repeatedly perform a high volume of short-duration and high-magnitude actions during competition [13].

The traditional threshold-based quantification of accelerations [14] has been criticized due to the high risk of overestimating accelerations initiated from low initial running speeds and underestimating those performed from high initial speeds (IntSs). Consequently, researchers have suggested incorporating the IntS to improve the accuracy of acceleration quantification [10,15,16,17]. In this context, recent methodologies have emphasized refined approaches to acceleration measurement to better capture the true physical demands of soccer match play. Therefore, assessing individual players accelerations capacity across different speed thresholds has become essential for monitoring training and match loads [10,15,16,17,18]. Currently, methodologies such as the acceleration–speed profile proposed by Morin et al. (2021) have been used to analyze sprint performance in real competitive contexts [18] and to examine the locomotor characteristics of high-intensity accelerations according to playing position [19]. Similarly, the novel acceleration–initial speed running (AS_0_) profile, based on the method of Sonderegger et al. (2016), offers an improved approach to quantify high-intensity accelerations [16,20]. This method provides clearer insight into maximal acceleration capacity across the entire velocity spectrum [16,17], and allows for individualized evaluation without the need for specific sprint tests from multiple IntSs [9,16,17,19].

In recent decades, researchers have highlighted the importance of positional roles and tactical formations in shaping competitive match demands [7,21,22]. Evidence indicates that external positions (e.g., wingers and full backs) are exposed to greater distances covered during high-speed running [7,21]. Moreover, players occupying these position roles demonstrate a greater frequency of accelerations initiated at high running velocities [16,19,23]. Conversely, central midfielders are exposed to greater acceleration demands and perform more actions initiated at low IntSs [16,19,23]. However, it remains unknown whether a player’s ability to accelerate at different IntSs influences the HIAs performed during competition.

Therefore, the aims of the present study were to (a) analyze the relationship between acceleration capacity at low and high initial speeds and the player’s ability to cover high-intensity running distances and perform high-intensity accelerations during competition and (b) examine whether these differences also occur across playing positions in professional soccer players.

We hypothesized that the relationship between acceleration capacity at low and high running speeds would influence the player’s ability to perform HIAs within different IntS intervals. Specifically, players with a greater acceleration capacity at a low IntS would demonstrate superior acceleration performance at low speeds, whereas players with greater acceleration capacity at a high IntS would exhibit greater acceleration performance at a high IntS and greater high-intensity running distances, irrespective of playing position.

## 2. Materials and Methods

### 2.1. Study Design

A retrospective, descriptive longitudinal study was designed to analyze the relationship between the maximal theoretical acceleration (A_0Int_) capacity and the maximal theoretical initial running speed (S_0Int_) regarding players capabilities to accelerate at different running speeds during competitive matches in the Spanish First Division during the 2021–2022 and 2022–2023 seasons.

### 2.2. Subjects

A total of 222 professional male soccer players from the Spanish First Division (LALIGA) participated in the study during the 2021–2022 and 2022–2023 seasons. The sample included players who completed a minimum of 90 min during a match [24,25] and participated in at least 12 matches over the season. Soccer players were classified by the Mediacoach system into six groups, according to the position role in the match. However, several players were used in multiple positions across the matches, and the classifications were adjusted to correspond to their starting position in each match. The descriptive statistics by position were based on all recorded appearances in each role: central defender (CD; n = 92, observations = 1745), full back or external defender (FB; n = 73, observations = 1023), central midfielder (CM; n = 60, observations = 735), offensive midfielder (OM; n = 52, observations = 321), wingers or external midfielder (W; n = 51, observations = 359), and forward (F; n = 32, observations = 371). The average number of match observations per player in 2021–2022 was 20.5 ± 6.37 and in 2022–2023 it was 20.5 ± 6.71. Goalkeepers were excluded from the study due to the different nature of their activity profile. The study received ethical approval from the Universidad Autónoma de Madrid (CEI-144-3233), in accordance with the latest version of the Declaration of Helsinki.

### 2.3. Procedures

Physical demands during a match were quantified using the TRACAB Gen5 optical player-tracking system (ChyronHego, New York, NY, USA). This system comprises 16 cameras (1920 × 1200 pixels) strategically positioned throughout the stadium, capturing player movements at a sampling frequency of 25 Hz. The TRACAB Gen5 system computes three-dimensional positional data, including horizontal (x, y) and vertical (z) coordinates, for each player in real time. Instantaneous speed was derived as the rate of change in the two-dimensional player position (XY plane) over time, while acceleration was subsequently calculated as the rate of change in speed over time. Raw tracking data were post-processed using the Mediacoach platform (La Liga, Madrid, Spain), which applies proprietary data-correction algorithms to enhance the accuracy and reliability of the exported physical performance variables [6,26].

The validity of the TRACAB Gen5 system for analyzing movement demands during soccer matches was established using a reference optical tracking system (VICON motion system, Oxford, UK). The TRACAB Gen5 system demonstrated root mean square errors (RMSEs) of 0.08 m for distance, 0.08 m·s^−1^ for speed, and 0.21 m·s^−2^ for acceleration during specific match-related actions. The 95% error ranges indicated positional errors between 0.02 and 0.15 m, speed errors between −0.10 and 0.09 m·s^−1^, and acceleration errors between −0.21 and 0.21 m·s^−2^ [26]. Previous studies have proven the validity and reliability of this instrument to assess running distances at different speeds [5]. More recently, TRACAB Gen5 has also been validated against GPS-IMU devices, showing very high consistency between both systems for moderate-intensity accelerations (2–4 m·s^−2^), with an explained variance of R^2^ = 0.95. However, the agreement was substantially lower for high-intensity accelerations (>4 m·s^−2^), where the relationship decreased to R^2^ = 0.338. In this acceleration range, TRACAB Gen5 tends to overestimate the number of accelerations, whereas GPS-IMU systems tend to underestimate them. Overall, the TRACAB optical tracking system, together with the Mediacoach software, can be considered a valid and reliable method for quantifying the number of accelerations, although it should not be used interchangeably with GPS-IMU systems [6].

### 2.4. Variables and Data Analysis

AS_0_ profiles from competition were analyzed for every match observation included in this study. Maximal acceleration values from each IntS were obtained from the dataset. Before creating the AS_0_ profiles of each player, the data was filtered and processed using the “density-based clustering algorithm” [27], which allowed for removing values without neighbors for each player. After removing outliers, the AS_0_ profile during competition was analyzed for each player in both seasons (2021–2022 and 2022–2023). For every 5-km·h^−1^ speed sub-interval, the maximal acceleration value was extracted and used for subsequent analyses. Each player’s AS_0_ profile was characterized by two main variables [18]: A_0Int_ (maximal theoretical acceleration) and S_0Int_ (maximal theoretical initial running speed).

High-intensity accelerations were quantified using the AS_0_ profile derived from all professional soccer players competing in LALIGA, establishing an absolute population-level threshold [16]. Specifically, a line parallel to 75% of the mean AS_0_ profile (Mean_75_AS_0_) was calculated, and all accelerations exceeding this threshold were classified as high-intensity accelerations. In addition, accelerations were further categorized into four groups according to IntS: 0–7, 7–14, 14–21 and >21 km·h^−1^. To evaluate the match physical performance, eight dependent variables were examined:

- Accelerations above 75% of the mean AS_0_ profile in LALIGA: The number of accelerations performed by the player above the Mean_75_AS_0_ profile [16].
- Accelerations 0–7 km·h^−1^ (Acc 0–7 km·h^−1^): The number of accelerations performed by the player above the Mean_75_AS_0_ profile within the 0 to 7 km·h^−1^ speed zone.
- Accelerations 7–14 km·h^−1^ (Acc 7–14 km·h^−1^): The number of accelerations performed by the player above the Mean_75_AS_0_ profile within the 7 to 14 km·h^−1^ speed zone.
- Accelerations 14–21 km·h^−1^ (Acc 14–21 km·h^−1^): The number of accelerations performed by the player above the Mean_75_AS_0_ profile within the 14 to 21 km·h^−1^ speed zone.
- Accelerations > 21 km·h^−1^ (Acc > 21 km·h^−1^): The number of accelerations performed by the player above the Mean_75_AS_0_ profile above 21 km·h^−1^.

Moreover, to characterize the match speed profile associated with running performance, three distance-based variables were analyzed:

- High-speed running (HSR, 21–24 km·h^−1^): Distance covered during the match when running at between 21 km·h^−1^ and 24 km·h^−1^.
- Very high-speed running (VHSR, >24 km·h^−1^): Distance covered during the match when running at >24 km·h^−1^.
- Sprinting (SPRINT, >28 km·h^−1^): Distance covered during the match when running at >28 km·h^−1^.

This study included five contextual variables:

- Own team ranking: The ranking of the player team at the end of the season were divided into four groups. In the upper table (UT), teams were grouped from first to fifth place; in the upper-middle table (UMT), teams were grouped from fifth to tenth place; in the lower-middle table (LMT), teams were grouped from eleventh to fifteenth place; and in the lower table (LT), teams were grouped from sixteenth to twentieth place.
- Opponent team ranking: The ranking of the opponent team at the end of the season were divided into four groups. In the upper table (UT), teams were grouped from first to fifth place; in the upper-middle table (UMT), teams were grouped from fifth to tenth place; in the lower-middle table (LMT), teams were grouped from eleventh to fifteenth place; and in the lower table (LT), teams were grouped from sixteenth to twentieth place.
- Match location: Home matches versus away matches.
- Match result: Match results were categorized into three categories: win, draw and loss.
- Congested period: Number of matches played in the same week. Congested: the players play two matches (minimum of 90 min in the match) in the same week. Non-congested: the players play one match (minimum of 90 min in the match) in the same week.

### 2.5. Statistical Analysis

First, linear mixed models (LMMs) accounting for intra-subject variability were employed to determine the minimum match requirements necessary to obtain a reliable and robust AS_0_ profile. For this analysis, only players who had participated in more than 20 matches were included, and their profiles were compared across cumulative match thresholds of 1, 3, 5, 8, 10, 12, 15, 20, and more than 20 matches. The goodness of fit of the linear relationship between the acceleration capacity and initial speed was assessed using the coefficient of determination (R^2^). To verify statistical assumptions, histograms and quantile–quantile (Q–Q) plots of the residuals were generated to evaluate the normality of the random effects. Pairwise comparisons of the estimated means were performed using Tukey’s method. Statistical significance was established for *p*-value < 0.05. This criterion was used to select the study participants (Supplementary Files).

K-means clustering was applied to classify the dataset into distinct groups based on two AS_0_ profile variables: A_0Int_ and S_0Int_. Prior to applying K-means clustering, a winsorization procedure was implemented to mitigate the influence and reducing the risk of extreme values [28]. After applying the winsorization procedure, A_0Int_ and S_0Int_ were standardized prior to conducting the K-means clustering. This ensured that the clustering algorithm operated on a uniform scale. The optimal number of clusters was determined using the elbow method, which suggested either 3 or 4 as suitable options. The evaluation of clustering performance using the silhouette coefficient, Davies–Bouldin Index, and Calinski–Harabasz Index indicated that the four-cluster model provided a superior overall fit compared with the three-cluster alternative. Specifically, the four-cluster model achieved a higher silhouette coefficient (0.363 vs. 0.360), reflecting improved internal cohesion and separation. This was further supported by a lower Davies–Bouldin Index (0.942 vs. 1.038), suggesting reduced cluster overlap. Additionally, the Calinski–Harabasz Index was higher for the four-cluster solution (165.981 vs. 151.404), indicating better-defined and more distinct cluster structures. Collectively, these metrics support the selection of a four-cluster configuration as the optimal model.

All dependent variables satisfied the assumption of normality, as assessed through histograms, Q–Q plots, skewness, and kurtosis, except for the ACC > 21 km·h^−1^ and SPRINT variables. Linear mixed models were employed to examine the effects of cluster membership and playing position (fixed effects) on the acceleration and running performance metrics, with player identity included as a random effect to account for the repeated-measures structure of the data. Contextual variables, including team ranking, opponent ranking, match location, match result, and fixture congestion, were incorporated as additional fixed effects. To verify the statistical assumptions, histograms and Q–Q plots of the residuals were generated to evaluate the normality of the random effects, and residual plots were inspected to assess the homoscedasticity. Given the violation of normality, a generalized linear mixed model (GLMM) was applied for the ACC > 21 km·h^−1^ and SPRINT variables. Consequently, coefficients (β) for these variables are expressed on a log-scale. Two model specifications were compared for each outcome: a full model including interaction effects between cluster membership and playing position, and a reduced model without this interaction term. Model selection was based on the Akaike Information Criterion (AIC), the coefficient of determination (R^2^), the root mean square error (RMSE), and the likelihood ratio test (LRT) [29]. Pairwise comparisons of estimated marginal means were conducted using Tukey’s method. Statistical significance was set at *p* < 0.05.

Finally, a chi-square test (*χ*^2^) was used to evaluate the association between positional role and cluster. As some players performed in multiple positional roles across the matches, each positional role was included independently in the statistical analysis. Moreover, a chi-square test was used to evaluate the association between the team ranking and cluster, the team ranking was evaluated in relation to the player’s final team of the season. Statistical significance was established for a *p*-value < 0.05. All statistical procedures were carried out using RStudio (version 4.5.2 2009–2026; RStudio, PBC, macOS).

## 3. Results

Statistical analysis determined a minimum of 12 matches per player to obtain a reliable and robust AS_0_ profile, as no statistically significant differences were observed between 12 and 15 matches (A_0Int_: *p* = 1.000; S_0Int_: *p* = 0.862), 12 and 20 matches (A_0Int_: *p* = 0.998; S_0Int_: *p* = 0.230), or 12 and >20 matches (A_0Int_: *p* = 0.465; S_0Int_: *p* = 0.054). Consequently, only players who had participated in more than 12 matches per season were retained for subsequent analyses. After applying this criterion, the initial sample of 470 players was reduced to 222 players. The AS_0_ profile demonstrated a nearly perfect linear fit across all players (R^2^ = 0.985 ± 0.012).

**Figure 1:**
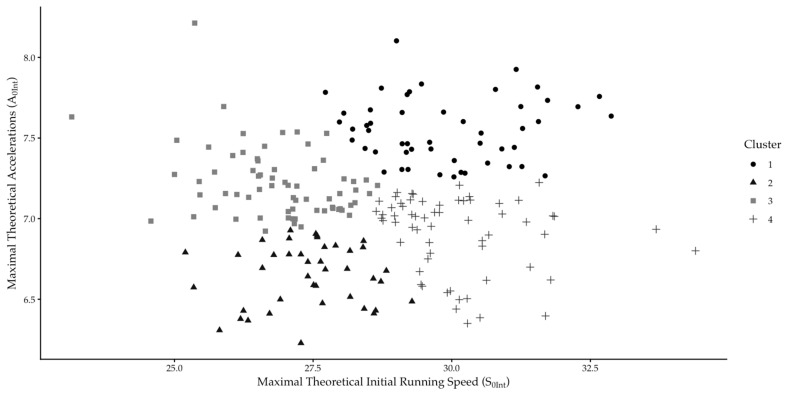
Illustration of the cluster groups based on the relationship between A_0Int_ and S_0Int_ in professional soccer players in LALIGA.

**Figure 2:**
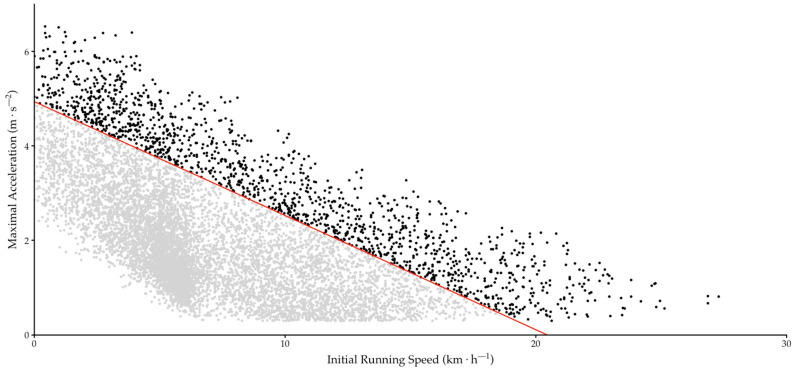
Example of a player’s accelerations derived from the Mean_75_AS_0_ in LALIGA.

Cluster analysis was subsequently employed to group players according to their acceleration capacity, as characterized by the AS_0_ profile, based on the interaction between the acceleration capacity at a low initial running speed (A_0Int_) and the acceleration capacity at high initial running speed (S_0Int_). This analysis yielded four distinct clusters: Cluster 1 (C1), comprising players with a high A_0Int_ and high S_0Int_; Cluster 2 (C2), comprising players with a low A_0Int_ and low S_0Int_; Cluster 3 (C3), comprising players with a high A_0Int_ and low S_0Int_; and Cluster 4 (C4), comprising players with a low A_0Int_ and high S_0Int_. A visual representation of the cluster distribution based on the A_0Int_–S_0Int_ relationship is presented in Figure 1. A graphical representation of accelerations performed above the Mean_75_AS_0_ threshold is displayed in Figure 2.

Table 1 shows the estimated coefficients, standard errors and the statistical differences across models for the acceleration-related variables. Regarding cluster effects, C1 displayed significantly higher coefficients for the TOTAL ACC and accelerations performed at low initial running speeds (ACC 0–7 km·h^−1^) compared with all other clusters and for accelerations at moderate initial running speeds (ACC 7–14 km·h^−1^) compared with C2. For accelerations performed at higher initial running speeds (ACC 14–21 km·h^−1^), C4 exhibited marginally significantly higher coefficients relative to the remaining clusters. With respect to ACC > 21 km·h^−1^, players belonging to C1 performed significantly more accelerations than those in C2 and C3. Regarding playing position, a significant effect was observed across the total accelerations and all acceleration categories stratified by IntS. Specifically, FB, CM, W, F and OM demonstrated significantly higher coefficients compared with CD.

Table 2 presents the estimated coefficients, standard errors, and statistical differences across models for high-intensity running metrics. Regarding cluster effects, C1 displayed significantly higher coefficients for HSR, VHSR, and SPRINT distance compared with all other clusters, except for HSR, for which C4 exhibited a marginally higher coefficient relative to the remaining clusters. A significant effect of playing position was observed across all running variables. For HSR, CD demonstrated significantly lower coefficients than all other playing positions. With respect to VHSR, CD showed significantly lower coefficients compared with F, FB, OM, and W. Similarly, for sprint distance, CD exhibited significantly lower coefficients relative to F, FB, and W.

Concerning contextual variables, the results presented in Table 1 and Table 2 revealed no statistically significant effect of team ranking on acceleration or running performance metrics, with the exception of ACC > 21 km·h^−1^, for which a significantly or marginally significantly higher number of accelerations was observed relative to the other clusters. In contrast, opponent ranking yielded statistically significant effects, with players exhibiting greater physical demands when competing against UT teams. Specifically, significantly higher coefficients were observed for TOTAL ACC, ACC 7–14 km·h^−1^, ACC 14–21 km·h^−1^ and ACC > 21 km·h^−1^, with the latter two showing significantly higher coefficients exclusively when comparing UT against LMT teams. With respect to high-intensity running metrics, greater physical demands were similarly observed when facing UT opponents, with significantly higher values recorded for HSR distance across all clusters. For VHSR, significant differences were restricted to comparisons between UT and MT teams. Match location also produced noteworthy findings, with away matches associated with significantly lower values across all HIAs variables compared with home matches, except for SPRINT distance. Finally, during congested competitive periods, players demonstrated significantly or marginally significantly lower values for TOTAL ACC, ACC 14–21 km·h^−1^, ACC 14–21 km·h^−1^ and HSR.

**Figure 3:**
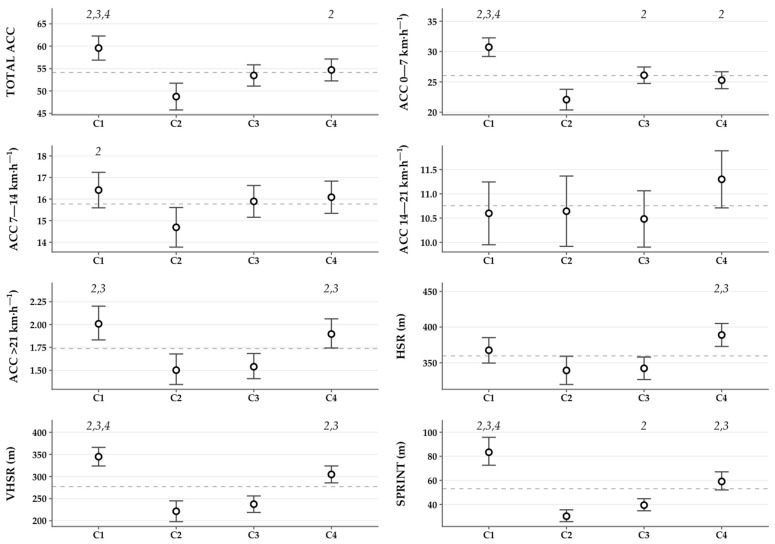
Estimated marginal means and confidence intervals of TOTAL ACC, ACC 0–7 km·h^−1^, ACC 7–14 km·h^−1^, ACC 14–21 km·h^−1^, ACC > 21 km·h^−1^, HSR, VHSR and SPRINT across cluster groups. 1—Substantial differences with cluster C1; 2—Substantial differences with cluster C2; 3—Substantial differences with cluster C3; 4—Substantial differences with cluster C4.

Figure 3 illustrates the estimated marginal means (EMMs) and 95% confidence intervals (95% CI) for acceleration variables across initial running speed categories and high-intensity running metrics, stratified by cluster group. For total accelerations, C1 recorded the highest value (EMM = 59.58; 95% CI: 56.89–62.28), with significantly greater values than all other clusters, followed by C4 (EMM = 54.69; 95% CI: 52.24–57.14), which also demonstrated significantly higher values than C2. Similar patterns were observed for accelerations at low initial running speeds (ACC 0–7 km·h^−1^), where C1 (EMM = 30.72; 95% CI: 29.19–32.26), C3 (EMM = 26.10; 95% CI: 24.74–27.46), and C4 (EMM = 25.27; 95% CI: 23.88–26.67) all exhibited significantly higher values than C2. For accelerations at moderate initial running speeds (ACC 7–14 km·h^−1^), C1 (EMM = 16.42; 95% CI: 15.59–17.24) demonstrated significantly greater values than C2. Regarding high-intensity accelerations (ACC > 21 km·h^−1^), C1 (EMM = 2.01; 95% CI: 1.83–2.20) and C4 (EMM = 1.90; 95% CI: 1.74–2.06) recorded significantly higher values than both C2 and C3. With respect to high-intensity running metrics, C4 recorded the highest HSR distance (EMM = 388.87 m; 95% CI: 372.72–405.01 m), with significantly greater values compared with C2 and C3. For the VHSR distance, C1 (EMM = 344.78 m; 95% CI: 323.71–365.86 m) and C4 (EMM = 304.71 m; 95% CI: 285.55–323.86 m) demonstrated the highest values, with both being significantly greater than the remaining clusters. A similar pattern was observed for sprint distance, where C1 (EMM = 83.35 m; 95% CI: 72.56–95.75 m) recorded the greatest value, significantly exceeding all other clusters, followed by C4 (EMM = 59.08 m; 95% CI: 52.05–67.06 m).

**Figure 4:**
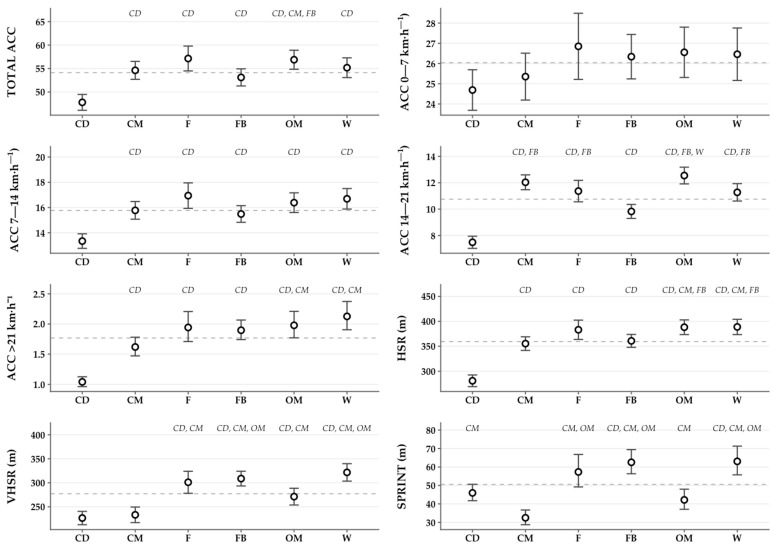
Estimated marginal means and confidence intervals of TOTAL ACC, ACC 0–7 km·h^−1^, ACC 7–14 km·h^−1^, ACC 14–21 km·h^−1^, ACC > 21 km·h^−1^, HSR, VHSR and SPRINT across position roles. CD—Substantial differences with central defender; CM—Substantial differences with central midfielder; F—Substantial differences with forward; FB—Substantial differences with full back; OM—Substantial differences with offensive midfielder; W—Substantial differences with winger.

Figure 4 represents the EM and 95% CI of variables related to the running metrics and accelerations according to positional role.

A chi-square test (χ^2^) revealed a statistically significant association between the playing position and cluster membership (χ^2^ = 68.184, df = 15, *p* < 0.001), indicating that the distribution of AS_0_ profiles across clusters was not random and that certain positional roles demonstrated a greater affinity for specific acceleration patterns. In contrast, no statistically significant association was found between cluster membership and the team’s final league ranking (χ^2^ = 16.36, df = 12, *p* = 0.175). With respect to positional distribution within clusters, CD showed the largest proportion of players in C3 (42.4%), reflecting a predominantly force-oriented mechanical profile characterized by a higher acceleration capacity at a low IntS. A similar pattern was observed among FB, with the largest proportions recorded in C1 (34.3%) and C3 (35.6%), suggesting a mixed mechanical profile combining both force- and velocity-oriented acceleration capacities. Among F, players were predominantly classified within C4 (40.6%), followed by C1 (25.0%), C3 (25.0%), and C2 (9.4%). CM and OM showed the largest proportions in C2 (38.3% and 32.7%, respectively), reflecting lower overall acceleration capacity. Finally, W showed the highest proportion of players in C4 (47.1%), consistent with a velocity-oriented profile characterized by superior acceleration capacity at a high IntS (Table 3).

## 4. Discussion

The primary objective of the present study was to examine the relationship between sprint capabilities derived from accelerations at different IntSs and the HIAs performed during official LALIGA matches. Cluster analysis enabled the categorization of players according to their acceleration capacities across a range of running speeds, providing insight into individual player characteristics for performing HIAs during competition. Players were grouped based on their sprint capacity orientation, distinguishing between those with predominant acceleration capacity at a low IntS, reflecting a force-oriented profile, and those with acceleration capacity at a high IntS, reflecting a velocity-oriented profile, grouping players into four clusters. Specifically, C1 was characterized by a high overall sprint capacity across all running speeds, C2 by a low overall sprint capacity, C3 by high acceleration capacity at a low IntS (i.e., a force-oriented profile), and C4 by a high acceleration capacity at a high IntS (i.e., a velocity-oriented profile) [30].

The primary findings indicate that cluster analysis revealed consistent and coherent AS0 profiles aligned with player’s acceleration capacities across low and high IntSs. A player characterized by high sprint capabilities (C1) demonstrated the most complete profile, performing a significantly greater number of HIAs across all IntSs, except for accelerations between 14 and 21 km·h^−1^. Furthermore, players in C1 covered greater distances during HSR, VHSR and SPRINT compared with players characterized by a low sprint capacity (C2). This mechanical profile was associated with an enhanced ability to perform HIAs under competitive conditions, with translation into superior match-play physical output. Players in C2, characterized by low sprint capacity, performed a significantly lower accelerations exceeding the Mean_75_AS_0_ threshold during competition compared with players in all other clusters across all IntS categories (0–7 km·h^−1^, 7–14 km·h^−1^, 14–21 km·h^−1^ and >21 km·h^−1^). These players also covered significantly shorter distances during HSR, VHSR and SPRINT. Players in C3, characterized by high acceleration capability at a low IntS, demonstrated a high level of performance in accelerations initiated at a low IntS (0–7 km·h^−1^ and 7–14 km·h^−1^); however, no statistically significant differences were observed between C3 and C4 for these variables. Players in C4, characterized by high acceleration capability at a high IntS, demonstrated a high level of performance in accelerations initiated at a high IntS (14–21 km·h^−1^ and >21 km·h^−1^). Furthermore, players in C1 and C4 covered significantly greater distances during HSR, VHSR, and SPRINT compared with players in C2 and C3. This finding may be attributable to the mechanical characteristics of sprint efforts, as most actions exceeding 25.2 km·h^−1^ rarely originate from a static position but instead are initiated from a high IntS [20]. In contrast, players in C1 and C3 performed significantly higher accelerations at a low IntS, which may be attributable to their greater force-oriented acceleration capacity [31]; however, no statistically significant differences were observed between C3 and C4 for these variables.

These findings confirm that characteristics of HIAs during competition are highly position-specific [19,32,33], except for accelerations between 0–7 km·h^—1^, where no statistically significant differences were found. However, our analysis also demonstrated that these characteristics vary according to a player’s sprint capacity, particularly among those with low capacities at both low and high IntSs. Among CD, despite a high proportion of players being classified within C3 (42.4%), this positional role performed significantly fewer accelerations than all other playing positions. This may suggest that even when players possess a high acceleration capacity, CDs are exposed to comparatively lower acceleration demands during competitive match play, indicating that positional tactical constraints may limit the expression of their acceleration capacity. Consequently, accelerations initiated at a low IntS appear to represent a key performance component for this positional role in achieving higher HIAs outputs during competition. Furthermore, CD demonstrated significantly shorter distances covered during HSR, VHSR, and SPRINT compared with all other positional roles. This trend is consistently supported by previous research, which reports lower HSR and SPRINT distances for CD relative to players in other positions [34], as well as substantially lower accelerations [19,23]. These results are in line with the positional demands inherent to the CD role, which is characterized by a greater proportion of time spent in standing or low-speed locomotion [35], with sprint efforts predominantly occurring in reactive defensive situations, such as covering runs and tracking opponents when out of ball possession [36]. Under these constraints, accelerations are more frequently initiated from low initial running speeds, which is consistent with the force-oriented profile observed in the largest proportion of CD players (C3, 42.4%) in the present study.

Among FB, the largest proportion of players was classified within C1 (34.3%) and C3 (35.6%), suggesting that optimal performance in this position requires high acceleration capacity across both low and high IntSs, respectively. Nevertheless, despite this mixed mechanical profile, FB demonstrated lower accelerations compared with other positional roles, which may reflect the combined defensive and offensive positional demands that may limit the frequency of high-intensity accelerative actions during match play. However, although the number of accelerative efforts may be lower, the distance covered and duration of each individual action appear to be greater [19], as evidenced by the significantly higher distances recorded during VHSR and SPRINT compared with other positional roles. This suggests that FB is not primarily characterized by a high frequency of short explosive accelerations, but rather by sustained HIAs initiated from a high IntS, which is consistent with the greater VHSR distances observed in this positional role [20].

Central and attacking positional roles, including OM, F, and W, demonstrated the highest acceleration and high-intensity running demands during competition [10,19,23], with F and W particularly distinguished by their elevated distances covered during HSR, VHSR, and SPRINT. The nature of their tactical role, which primarily involves explosive offensive movements such as sprinting into space, making sharp cuts to receive passes, running with the ball or transitions [36,37], could increase the effort required at high intensity during the match. These positional roles were collectively characterized by a large representation of players across C1, C3, and C4, reflecting diverse physical demands associated with central and attacking positions in professional soccer. This distribution suggests that players occupying these roles must possess the capacity to perform HIAs across a wide range of IntSs, encompassing both force-oriented and velocity-oriented accelerative actions, to meet the varied tactical and physical requirements of their positional roles during competitive match play [36,37]. Moreover, when analyzing descriptive data of the total distance covered during HSR, wider positional roles (such as W and FB), followed by offensive central positional roles (such as F and OM) showed a higher distance covered during HSR, VHSR and SPRINT than defensive central positional roles (such as CM and CD) [33,38], according to previous studies that show a higher distance covered during high-intensity running in the Croatian First Division [33], Spanish LALIGA [8,39,40], English Premier League [37,39], international football tournaments [7] and European competitions [21]. Moreover, previous studies have also reported that wider positional roles perform a higher number of high-intensity accelerations (>3 m·s^−2^) compared with central positions [10,32,41]. In this regard, in contrast with previous studies, central positions such as CMs and OMs at moderate IntS (14–21 km·h^−1^) showed higher acceleration values relative to other positions.

These findings illustrate that identifying the acceleration capacity at specific IntS thresholds in professional soccer provides players with a more accurate estimation and characterization of the physical demands during competition, providing deeper insight into the levels of HIAs that players can achieve during a match. The highest total accelerations outputs were observed in players classified within C1, C3 and C4, whereas greater distances covered during high-intensity running were in C1 and C4, highlighting the influence of both force-oriented and velocity-oriented profiles on match-play physical output. These findings reinforce the importance of assessing individual acceleration capacity across the full range of IntS thresholds to better characterize the specific physical and conditional demands during competition.

Finally, contextual factors significantly influenced HIAs. Match location was a particularly relevant factor across nearly all variables, with performance generally reduced in away matches, with the sole exception of the SPRINT distance. However, the literature presents considerable controversy regarding match demands as a function of match location. On one hand, González-Rodenas et al. (2024) [42] reported comparable reductions in distances covered at 21–24 km·h^−1^ and above 24 km·h^−1^ during away fixtures in LALIGA [42], with similar observations reported at the fourth division level in Spain [43]. On the other hand, studies conducted in the Premier League found that HSR and SPRINT distances without ball possession were greater in away matches [37], a finding complemented by higher accelerations and decelerations without ball possession, as well as decelerations during ball possession, also being greater away from home [44]. Furthermore, higher maximal deceleration values in away matches were reported [45], whereas home matches were associated with greater maximal acceleration values and distance gained [19,45]. These discrepancies across studies may reflect differences in competitive level, tactical context, and offensively and defensively oriented strategies characterized by reduced ball possession time. Regarding the influence of competitive level, team ranking did not yield statistically significant effects on HIA outputs across most variables, except for ACC > 21 km·h^−1^, for which players belonging to UT teams demonstrated significantly and marginally higher performance values. In contrast, matches played against higher-ranked opponents were associated with increased overall match demands, consistent with previous research in professional soccer players [19,37,44]. With respect to match results, a significant or marginal influence on overall match demands was observed, which showed increased demands in matches where the team ultimately lost. As with the variables discussed above, the existing literature presents discrepant findings on this topic. Nevertheless, a general trend appears to emerge whereby matches ending in a draw are associated with lower physical output, whereas losing scorelines tend to elicit greater HIAs [45]. It should be noted, however, that match status must be carefully considered in this context, as it can substantially alter a team’s tactical approach and playing style, and consequently modify match physical demands in a complex and dynamic manner [46].

### 4.1. Limitations

The present study has some limitations that need to be acknowledged. First, the methodology for evaluating the AS_0_ profile is not carried out with a sprint test at different initial constant sprints, although it has been evaluated using the data provided by multi-camera tracking system. However, we know that maximal acceleration can be questioned, and therefore, we included players who completed a minimum of 90 min in the match and participated in at least 12 matches over the season when assessing the maximal acceleration capacities. Second, the positional roles were assigned by the data provider, which was assigned automatically based on the position in which they spent the majority of the playing time. Third, the analysis of congested fixture periods may be constrained by the definition of the time window used to classify matches as congested, given that different thresholds could yield divergent results. Finally, match-specific player data may affect the assessment of maximum acceleration capacity, as some players participated in only a single match during the season.

### 4.2. Future Research

Future research should consider integrating technical–tactical factor analyses of offensive and defensive performance indicators to better contextualize HIAs in competition. This approach would enhance the characterization of the AS_0_ profile. Moreover, exploring the incorporation of deceleration analysis may be beneficial, as these actions are key performance variables in soccer and occur as frequently as accelerations, increasing the contribution to the player’s mechanical load. Furthermore, future research could also examine individual maximal acceleration capacity by applying relative AS_0_ profile thresholds, enabling the identification of high-intensity accelerations exceeding >75% of the maximal acceleration capacity from different constant initial running speeds. Moreover, future research should aim to validate the AS_0_ profile using maximal sprint tests from different constant initial running speeds. Finally, future analysis could explore whether players whose characteristics in competition differ from the average positional demands influence tactical decision (e.g., OMs may drift more toward the external position, thereby increasing the demands for accelerations initiated at a high IntS).

### 4.3. Practical Applications

The cluster analysis method provides strength and conditioning coaches a practical tool to characterize acceleration profiles during both low and high IntS running in soccer players, and to examine their relationship with the demands in competition, without the need to do field-based maximal sprint tests from different constant initial running speeds. This practical characterization enhances the knowledge of acceleration capacity regarding different IntS thresholds. The impact of these accelerations profiles on athletic performance and identifies specific acceleration types that may challenge players in competition. These approaches inform targeted physical preparation strategies in training, tailored to the positional demands of each athlete. Finally, the findings of this study may assist clubs in identifying the physical characteristics of each positional role in the top teams and determine which player profiles best fit specific positional demands and tactical principles. This insight can help clubs evaluate which players may adapt effectively to the team’s playing style or enhance team physical performance demands.

## 5. Conclusions

This study highlights how the accelerations quantified in relation to the IntS vary according to the relationship between A_0Int_ and S_0Int_ during competition and the player positional role, showing that it is not only the position of the player on the field is important but also the player’s acceleration capacity. The main result of this study shows that players with a high acceleration capacity at low and high IntSs performed higher accelerations and covered a greater distance during high-intensity running in competition compared with players with a low capacity at low and high IntSs. Moreover, the relationship regarding acceleration capacity at low and high initial running speeds characterizes the prevalence of efforts performed by soccer players, where players with a high acceleration capacity at a low IntS achieve higher values for accelerations at a low IntS and players with a high acceleration capacity at a high IntS achieve higher values for accelerations at a high IntS and achieved a greater distance during high-intensity running.

## Figures and Tables

**Table 1:** Estimated coefficients, standard errors and *p*-values for acceleration variables.

	TOTAL ACC			ACC 0–7 km·h^−1^			ACC 7–14 km·h^−1^			ACC 14–21 km·h^−1^			ACC > 21 km·h^−1^		
	Coeff	SE	*p*	Coeff	SE	*p*	Coeff	SE	*p*	Coeff	SE	*p*	Coeff	SE	*p*
Intercept	55.22	1.80	***	30.17	1.04	***	14.84	0.58	***	7.41	0.46	***	0.33	0.07	***
Cluster															
C1															
C2	−10.84	2.03	***	−8.65	1.15	***	−1.73	0.62	**	0.04	0.49	0.93	−0.29	0.07	***
C3	−6.12	1.77	***	−4.62	1.01	***	−0.52	0.53	0.33	−0.12	0.42	0.78	−0.27	0.06	***
C4	−4.89	1.81	**	−5.45	1.02	***	−0.33	0.54	0.54	0.70	0.42	†	−0.06	0.06	0.33
Position Role															
CD															
CM	6.83	1.06	***	0.66	0.66	0.32	2.43	0.42	***	4.55	0.33	***	0.44	0.06	***
F	9.35	1.47	***	2.16	0.92	*	3.60	0.57	***	3.88	0.46	***	0.62	0.07	***
FB	5.32	0.91	***	1.65	0.58	**	2.15	0.38	***	2.34	0.30	***	0.60	0.05	***
OM	9.10	1.14	***	1.86	0.72	**	3.05	0.46	***	5.06	0.37	***	0.64	0.07	***
W	7.39	1.19	***	1.77	0.75	*	3.35	0.48	***	3.79	0.39	***	0.71	0.07	***
Team Ranking															
1. UT															
2. UMT	−1.25	1.68	0.46	−0.81	0.95	0.40	−0.24	0.50	0.63	0.06	0.39	0.89	−0.11	0.06	†
3. LMT	−0.98	1.77	0.58	−0.90	1.00	0.37	−0.03	0.53	0.95	0.07	0.41	0.87	−0.11	0.06	†
4. LT	−1.25	1.86	0.50	−0.31	1.06	0.77	−0.62	0.56	0.27	−0.19	0.44	0.66	−0.13	0.06	*
Opponent Ranking															
1. UT															
2. UMT	−0.89	0.37	*	−0.16	0.24	0.51	−0.72	0.18	***	0.10	0.15	0.49	−0.05	0.03	0.16
3. LMT	−1.73	0.37	***	−0.09	0.25	0.72	−0.88	0.18	***	−0.61	0.15	***	−0.07	0.03	*
4. LT	−1.07	0.38	**	−0.06	0.25	0.81	−0.73	0.18	***	−0.24	0.15	0.12	−0.01	0.03	0.86
Match Location															
Home															
Away	−1.18	0.26	***	−0.58	0.17	***	−0.26	0.13	*	−0.28	0.11	**	−0.04	0.02	†
Match Result															
Win															
Draw	−0.33	0.33	0.32	−0.30	0.22	0.18	−0.03	0.16	0.85	−0.05	0.13	0.71	0.02	0.03	0.51
Loss	0.30	0.34	0.37	−0.10	0.22	0.66	−0.05	0.16	0.74	0.36	0.13	**	0.08	0.03	*
Congested Match															
Congested															
Not Congested	0.84	0.40	*	0.41	0.26	0.12	0.22	0.19	0.25	0.33	0.16	*	−0.06	0.04	†

Note: TOTAL ACC—total number of accelerations exceeding the Mean_75_AS_0_ threshold; ACC 0–7 km·h^−1^—total number of accelerations from 0 to 7 km·h^−1^; ACC 7–14 km·h^−1^—total number of accelerations from 7 to 14 km·h^−1^; ACC 14–21 km·h^−1^—total number of accelerations from 14 to 21 km·h^−1^; ACC > 21 km·h^−1^—total number of accelerations above 21 km·h^−1^; C1—Cluster 1; C2—Cluster 2; C3—Cluster 3; C4—Cluster 4; CD—central defender; CM—central midfielder; F—forward; FB—full back; OM—offensive midfielder; W—winger; 1. UT—upper table; 2. UMT—upper-middle table; 3. LMT—lower-middle table; 4. LT—lower table; Coeff—estimated coefficients; SE—standard error; *p*—*p*-value; †—marginally significant (<0.10); *—significant (<0.05); **—highly significant (<0.01); ***—very highly significant (<0.001). Coefficients for ACC > 21 km·h^−1^ are expressed in log-scale.

**Table 2:** Estimated coefficients, standard errors and *p*-values for running variables.

	HSR			VHSR			SPRINT		
	Coeff	SE	*p*	Coeff	SE	*p*	Coeff	SE	*p*
Intercept	294.06	12.10	***	303.88	14.36	***	4.43	0.10	***
Cluster									
C1									
C2	−28.14	13.35	*	−123.46	15.85	***	−1.01	0.11	***
C3	−25.19	11.63	*	−107.41	13.80	***	−0.75	0.09	***
C4	21.48	11.83	†	−40.08	14.03	**	−0.34	0.09	***
Position Role									
CD									
CM	74.34	7.87	***	6.70	9.33	0.47	−0.35	0.07	***
F	102.15	10.91	***	74.57	12.93	***	0.22	0.09	*
FB	79.85	6.93	***	82.23	8.22	***	0.31	0.06	***
OM	107.27	8.56	***	44.64	10.15	***	−0.09	0.08	0.26
W	107.87	8.97	***	95.12	10.63	***	0.32	0.07	***
Team Ranking									
1. UT									
2. UMT	−6.06	10.97	0.58	−5.04	13.02	0.70	−0.07	0.09	0.41
3. LMT	7.98	11.56	0.49	3.62	13.72	0.79	−0.09	0.09	0.33
4. LT	−12.22	12.22	0.32	−2.29	14.50	0.87	−0.00	0.10	0.97
Opponent Ranking									
1. UT									
2. UMT	−5.32	2.94	†	−6.10	3.48	†	−0.04	0.03	0.12
3. LMT	−17.55	3.01	***	−13.57	3.57	***	−0.05	0.03	†
4. LT	−10.46	3.05	***	−5.88	3.61	0.10	−0.02	0.03	0.44
Match Location									
Home									
Away	−4.95	2.11	*	−10.70	2.50	***	0.00	0.02	***
Match Result									
Win									
Draw	3.65	2.68	0.17	0.60	3.17	0.85	0.02	0.02	0.48
Loss	8.80	2.71	**	5.90	3.20	†	0.04	0.02	†
Congested Match									
Congested									
Not Congested	7.94	3.19	*	1.72	3.78	0.65	0.01	0.03	0.62

Note: HSR—high-speed running (21–24 km·h^−1^); VHSR—very high-speed running (>24 km·h^−1^); SPRINT—sprint (>28 km·h^−1^); VMAX—maximum speed reached during each match; C1—Cluster 1; C2—Cluster 2; C3—Cluster 3; C4—Cluster 4; CD—central defender; CM—central midfielder; F—forward; FB—full back; OM—offensive midfielder; W—winger; 1. UT—upper table; 2. UMT—upper-middle table; 3. LMT—lower-middle table; 4. LT—lower table; Coeff—estimated coefficients; SE—standard error; *p*—*p*-value; †—marginally significant (<0.10); *—significant (<0.05); **—highly significant (<0.01); ***—very highly significant (<0.001). Coefficients for SPRINT distance are expressed in log-scale.

**Table 3:** Distribution of number of players (n and %) according to positional role and cluster.

Position Role	Cluster												Total
	C1			C2			C3			C4			
	n	%	STDRES	n	%	STDRES	n	%	STDRES	n	%	STDRES	
CD	21	22.83%	0.31	17	18.48%	−0.27	39	42.39%	3.31	15	16.30%	−3.32	92
CM	4	6.67%	−3.09	23	38.33%	4.05	20	33.33%	0.83	13	21.67%	−1.54	60
F	8	25.00%	0.48	3	9.38%	−1.51	8	25.00%	−0.51	13	40.62%	1.37	32
FB	25	34.25%	2.92	5	6.85%	−3.05	17	23.29%	−1.18	26	35.62%	1.17	73
OM	5	9.62%	−2.28	17	32.69%	2.60	13	25.00%	−0.67	17	32.69%	0.46	52
W	15	29.41%	1.45	5	9.80%	−1.88	7	13.73%	−2.58	24	47.06%	2.87	51

Note: Data represents n and %. CD—central defender; CM—central midfielder; F—forward; FB—full back; OM—offensive midfielder; W—winger. STDRES—standardized residuals, standardized residuals greater than 2 or lower than −2 were interpreted as significant deviations from the expected frequencies (*p* < 0.05). C1—Cluster 1; C2—Cluster 2; C3—Cluster 3; C4—Cluster 4.

## Data Availability

The data presented in this study are available on request from the corresponding author. The data are not publicly available due to confidentiality of the data.

## Supplementary Materials

The following supporting information can be downloaded at: https://www.mdpi.com/article/10.3390/sports14070273/s1, Table S1: Estimated coefficients, standard errors and *p*-values for A_0Int_ and S_0Int_; Figure S1: Histograms, Q–Q plots of residuals, residual plots to assess homoscedasticity and Q–Q plots of random effects for A_0Int_; Figure S2: Histograms, Q–Q plots of residuals, residual plots to assess homoscedasticity and Q–Q plots of random effects for S_0Int_; Table S2: Pairwise comparisons of the estimated means were performed using Tukey’s method; Table S3: R^2^ values for AS_0_ profiles; Table S4: Estimated Marginal Means and 95% Confidence Intervals.

## Abbreviations

The following abbreviations are used in this manuscript:

A_0Int_	Maximal theoretical acceleration
ACC	Acceleration
AS_0_	Acceleration–initial speed
Mean_75_AS_0_	Accelerations performed above 75% of the acceleration–speed profile
C1	Cluster 1
C2	Cluster 2
C3	Cluster 3
C4	Cluster 4
CD	Central defender
CM	Central midfielder
F	Forward
FB	Full back
HSR	High-speed running (21–24 km·h^−1^)
HIAs	High-intensity actions
IntS	Initial speed
LT	Lower table
LMT	Lower-middle table
OM	Offensive midfielder
*p*	*p*-value
S_0Int_	Maximal theoretical running speed
SPRINT	Sprinting (>28 km·h^−1^)
STDRES	Standardized residuals
UMT	Upper-middle table
UT	Upper table
VHSR	Very high-speed running (>24 km·h^−1^)
Vmax	Maximum speed reached during each match
W	Winger
*χ*^2^	Chi-square test
