# Supplementary material for: Impact of Sprint Performance Characteristics Across Acceleration–Initial Speed Profiles in LALIGA
Source: Sports (Basel). 2026 Jul 1;14(7):273. doi: 10.3390/sports14070273 (PMC13416579; doi:10.3390/sports14070273)
Supplement: Supplementary file 1 [file sports-14-00273-s001.zip › sports-4330601-supplementary.pdf]

Supplementary Material

Table S1. Estimated coefficients, standard errors and p-values for A_{0Int} and S_{0Int}.

	A_{0Int}			S_{0Int}		
	Coeff	SE	*p*	Coeff	SE	*p*
Intercept	6.60	0.04	***	25.79	0.20	***
Matches						
1						
3	0.25	0.03	***	1.28	0.18	***
5	0.39	0.03	***	1.90	0.18	***
8	0.45	0.03	***	2.11	0.18	***
10	0.50	0.03	***	2.33	0.18	***
12	0.54	0.03	***	2.36	0.18	***
15	0.55	0.03	***	2.63	0.18	***
20	0.57	0.03	***	2.81	0.18	***
>20	0.61	0.03	***	2.92	0.18	***

Note: Coeff—estimated coefficients; SE—standard error; *p*—*p*-value; †—marginally significant (<0.10); *—significant (<0.05); **—highly significant (<0.01); ***—very highly significant (<0.001).

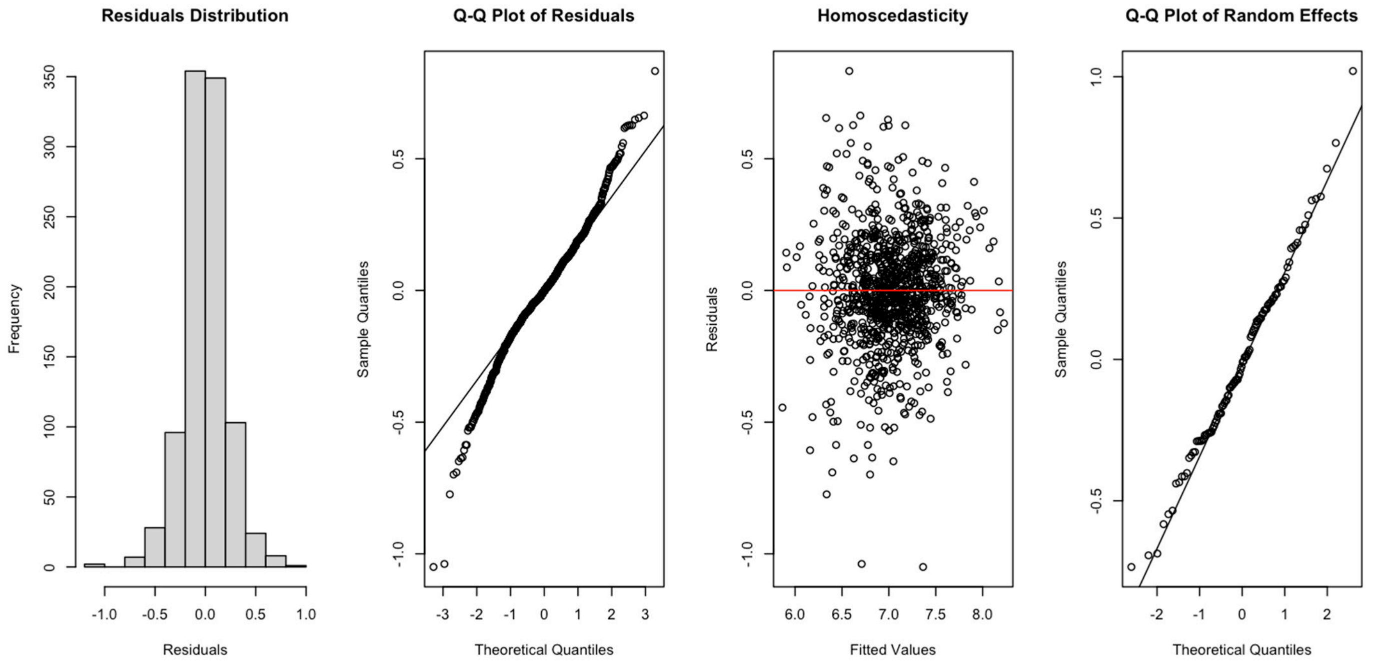

Figure S1. Histograms, Q–Q plots of residuals, residual plots to assess homoscedasticity and Q–Q plots of random effects for A_{0Int}.

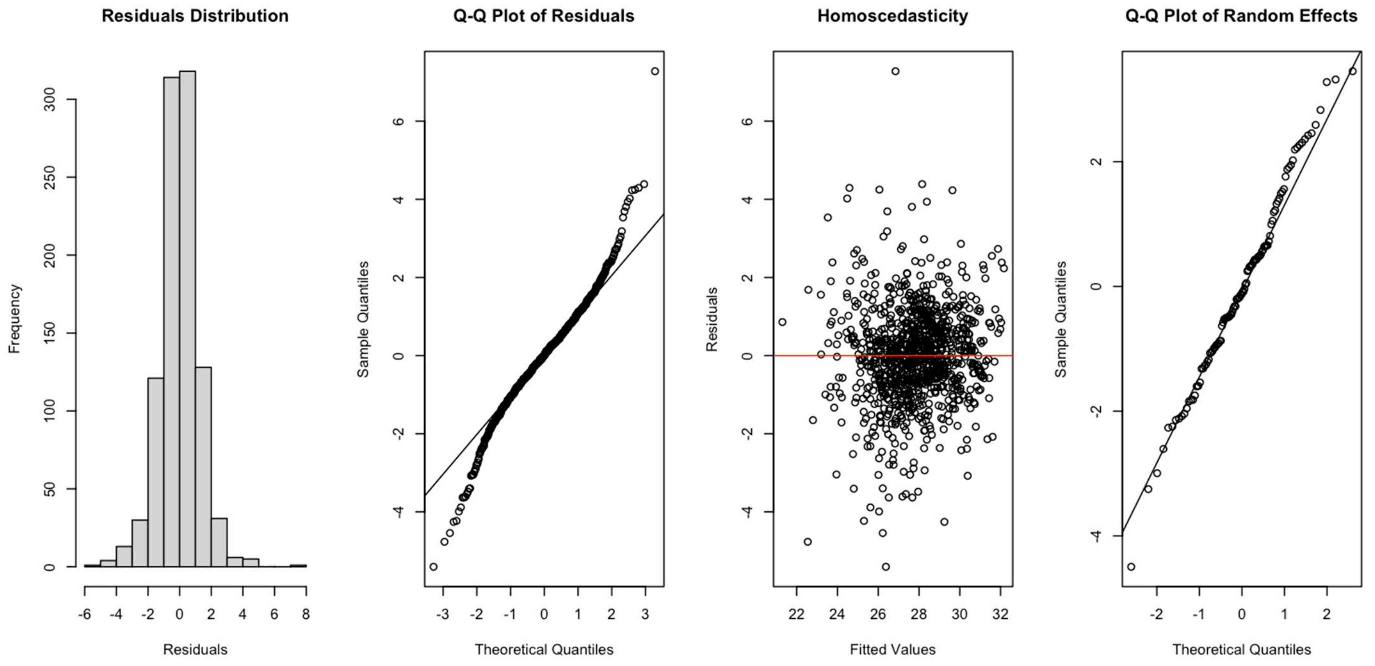

Figure S2. Histograms, Q–Q plots of residuals, residual plots to assess homoscedasticity and Q–Q plots of random effects for S_{0Int}.

Table S2. Pairwise comparisons of the estimated means were performed using Tukey's method.

	A_{0Int}	S_{0Int}
Match	*p*	*p*
1 vs 3	***	***
1 vs 5	***	***
1 vs 8	***	***
1 vs 10	***	***
1 vs 12	***	***
1 vs 15	***	***
1 vs 20	***	***
1 vs >20	***	***
3 vs 5	***	*
3 vs 8	***	***
3 vs 10	***	***
3 vs 12	***	***
3 vs 15	***	***
3 vs 20	***	***
3 vs >20	***	***
5 vs 8	0.558	0.961
5 vs 10	*	0.277
5 vs 12	***	0.191
5 vs 15	***	***
5 vs 20	***	***
5 vs >20	***	***
8 vs 10	0.889	0.948
8 vs 12	0.085	0.891
8 vs 15	*	0.089
8 vs 20	**	**
8 vs >20	***	***
10 vs 12	0.856	1.000
10 vs 15	0.661	0.768
10 vs 20	0.371	0.154
10 vs >20	**	*
12 vs 15	1.000	0.862
12 vs 20	0.998	0.230
12 vs >20	0.465	0.054
15 vs 20	1.000	0.984
15 vs >20	0.694	0.806
20 vs >20	0.913	1.000

Note: Coeff—estimated coefficients; SE—standard error; *p*—*p*-value; *—significant (<0.05); **—highly significant (<0.01); ***—very highly significant (<0.001).

Table S3. R^2 values for AS_0 profiles.

Matches	mean	sd	min	max
1	0.953	0.039	0.802	1.000
3	0.971	0.019	0.923	1.000
5	0.979	0.015	0.918	0.999
8	0.983	0.014	0.932	0.999
10	0.984	0.013	0.934	0.998
12	0.984	0.011	0.946	0.999
15	0.985	0.011	0.948	1.000
20	0.986	0.010	0.953	0.999
>20	0.986	0.009	0.947	0.999

Table S4. Estimated Marginal Means and 95% Confidence Intervals for A_{0Int} and S_{0Int}

	A_{0Int}			S_{0Int}		
Matches	EMM	Lower 95%CI	Upper 95%CI	EMM	Lower 95%CI	Upper 95%CI
1	6.60	6.52	6.67	25.79	25.40	26.18
3	6.85	6.77	6.92	27.07	26.68	27.46
5	6.99	6.92	7.07	27.69	27.30	28.08
8	7.05	6.98	7.13	27.91	27.52	28.30
10	7.10	7.02	7.17	28.13	27.74	28.52
12	7.14	7.07	7.22	28.16	27.77	28.55
15	7.15	7.08	7.23	28.43	28.04	28.82
20	7.17	7.09	7.24	28.61	28.22	29.00
>20	7.21	7.13	7.28	28.71	28.32	29.10

Note: EMM — Estimated Marginal Means; Lower 95% CI —Lower bound of the 95% Confidence Interval; Upper 95% CI — Upper bound of the 95% Confidence Interval.
